# Sanfu herbal patch applied at acupoints in patients with bronchial asthma: study protocol for a randomized controlled trial

**DOI:** 10.1186/s13063-020-04604-8

**Published:** 2020-07-29

**Authors:** Xiaoyan Xie, Danghan Xu, Lixing Zhuang, Hui Liu, Sui Tan, Yanqing Lu, Meiyi Su, Jie Chen, Haihua Pan, Lu Lu, Yiming Xu, Muxi Liao, Zhanqiong Xu, Jun He

**Affiliations:** 1 Guangzhou University of Chinese Medicine, Guangzhou, 510006 China; 2 Department of Rehabilitation Center, The First Affiliated Hospital of Guangzhou University of Chinese Medicine, Guangzhou, 510405 China; 3 The Fifth Affiliated Hospital of Guangzhou University of Chinese Medicine, Guangzhou, 510095 China; 4 Guangdong Second Provincial General Hospital, Guangzhou, 510317 China; 5 Pingshan District Peoples’ Hospital of Shenzhen, Shenzhen, 518118 China; 6 Lingnan Medical Research Center, The First Affiliated Hospital of Guangzhou University of Chinese Medicine, Guangzhou, 510405 China; 7 Guangzhou Medical University, Guangzhou, 515000 China

**Keywords:** Bronchial asthma, Sanfu herbal patch, Airway inflammation, RCT

## Abstract

**Background:**

Bronchial asthma is one of the most common inflammatory airway disorders. As one of the main non-drug therapies, the Sanfu herbal patch (SHP) has been widely used to treat bronchial asthma, although the evidence for its efficacy and associated mechanism are inconclusive. The objective of this trial is to clarify the clinical efficacy and safety of the SHP in the treatment of bronchial asthma in the chronic persistent or clinical remission stage and to provide high-quality data for further research.

**Methods:**

We propose a multicentre, double-blinded, parallel, randomized, placebo-controlled clinical trial involving 4 study hospitals in China. A total of 72 eligible participants will be randomized into an SHP group and a placebo group. They will receive an SHP for 3 treatment sessions. The primary outcome will be changes in forced expiratory volume in 1 s after 3 treatment sessions. Secondary outcomes will include the following: (1) the Asthma Quality of Life Questionnaire, Asthma Control Test, and Asthma Long-term Follow-up Scale; (2) levels of Metallothionein-2 and Transgelin-2 in blood and urine; and (3) levels of IL-5, IL-13, IL-23, IL-25, and thymic stromal lymphopoietin in blood. Analysis of the data will be performed at baseline, at the end of the 2nd and 3rd treatment sessions, and at the 24-week follow-up. The safety of the SHP will be evaluated at each treatment session.

**Discussion:**

The aims of this trial are to determine whether the SHP is more effective than placebo in the treatment of patients with bronchial asthma, as well as whether the SHP works by reducing airway inflammation and reversing bronchoconstriction.

**Trial registration:**

Chinese Clinical Trial Registry (http://www.chictr.org.cn), ChiCTR1900024616. Registered on 19 July 2019.

## Background

Bronchial asthma (BA) is one of the most common chronic inflammatory airway disorders, presenting with recurrent episodes of wheezing, recurrent cough, shortness of breath, sputum in the throat, and chest tightness [1]. Worldwide, more than 330 million people have BA [2], and the incidence of the disease has been increasing, with the incidence projected to reach 400 million by 2025 [3]. Among BA patients, Chinese BA patients now account for the largest proportion (36.7%) [4]. The high incidence and high recurrence rate of BA not only bring a heavy economic burden to patients but also seriously affect their quality of life. If symptoms persist, patients may have asthma exacerbations [5, 6], lung function decline [7], and pathological bronchial wall narrowing [8], which may cause psychological depression [9] and eventually lead to limited daily activities and even death [10].

Currently, the treatment for BA is to reduce symptoms and improve patients’ daily activities. The commonly used symptomatic pharmacotherapy options include desensitization therapy and drug treatments. The main drug treatments include inhaled corticosteroids (ICSs), beta-2 agonists, and leukotriene receptor antagonists (LTRAs) [11]. However, desensitization therapy has strict indications that limit its application [12]. Drug treatments could cause side effects, such as ulcers, Cushing’s syndrome, and osteoporosis, and the risk increases with age and treatment duration [13]. Therefore, a safe and effective therapy for BA is urgently required to improve patients’ quality of life and reduce uncontrollable symptoms and the national medical burden.

The Sanfu herbal patch (SHP) is important in traditional Chinese medicine as an external therapy for respiratory diseases [14, 15]. It has been widely used in China for more than 300 years [16]. The SHP is an herbal patch that is applied to specific acupoints to stimulate skin, meridians, and collaterals only during the Sanfu period to produce preventive and therapeutic effects. The Sanfu period (between mid-July and mid-August) is supposed to include the hottest days in the year in China. Asthma is the most common condition treated by the SHP [14]. The SHP is effective at reducing the frequency of symptoms, decreasing the need for concomitant medications, and improving lung function and quality of life [17, 18].

Although the SHP is widely used to treat BA and has achieved good results, there is still a lack of good medical evidence to verify whether it is beneficial. In addition, the molecular biological mechanism is still unclear. Studies have suggested that SHP has a positive effect on the immune outcomes of asthma [15], such as reducing interleukin 4 (IL-4) and significantly increasing IFN-γ [19].

A systematic review and meta-analysis of 34 randomized controlled trials (RCTs) published in 2017 involving 3313 participants showed that most of the studies were of poor quality [20]. Therefore, a full-scale and rigorously designed RCT that overcomes the identified methodological problems is necessary.

The motives for this study are twofold. The primary aim is to determine the clinical efficacy of SHP in BA patients after 3 treatment sessions (TSs) and a 24-week follow-up. Second, we hope to gain insight regarding whether the SHP works by reducing airway inflammation and reversing bronchoconstriction.

## Methods

### Trial design

**Fig. 1:**
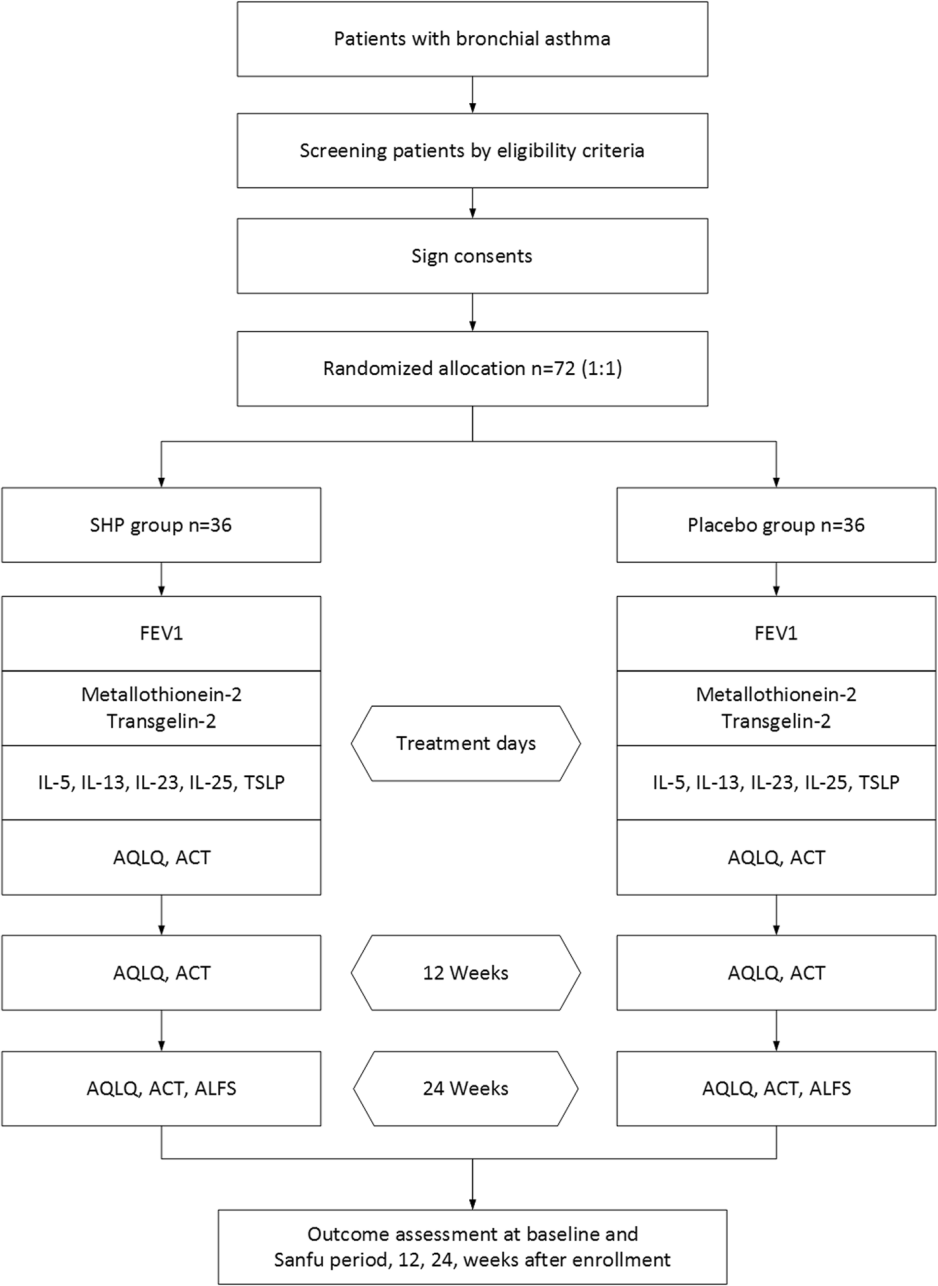
Trial flow chart. SHP, Sanfu herbal patch

**Fig. 2:**
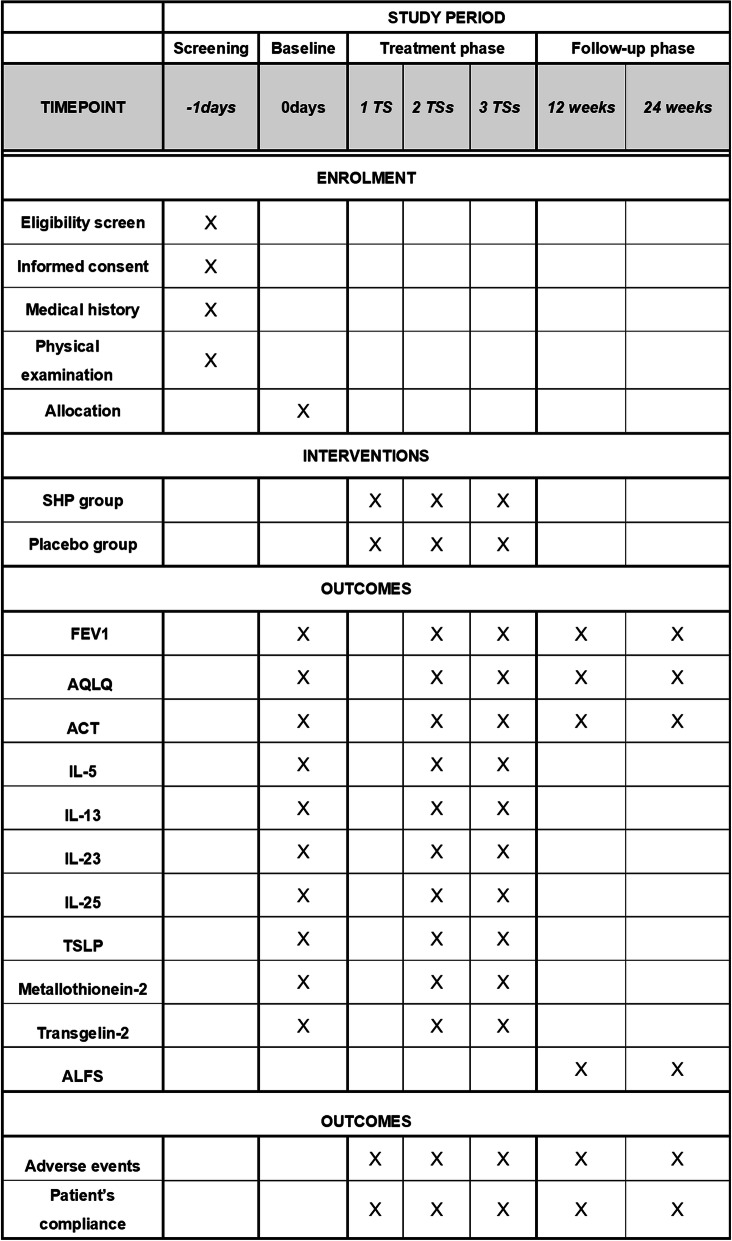
Study schedule. 1 TS: the 1st treatment during the first 10 days. 2 TS: the 2nd treatment during the second 10 days. 3 TS: the 3rd treatment during the third 10 days. 12 weeks: the first follow-up period from baseline. 24 weeks: the second follow-up period from baseline. All outcomes will be assessed at baseline, the end of 2 TS, and end of 3TS during treatment

This study is a multi-centre, parallel, randomized controlled trial. Participants with BA will be screened based on strict inclusion and exclusion criteria from January 2020 to December 2021. After providing written informed consent, 72 eligible participants will be randomly allocated to one of two groups: an SHP group receiving SHP treatment and a placebo group receiving placebo patch treatments. They will receive treatment for 3 TSs and follow-up assessments for 24 weeks (Figs. 1 and 2). The trial has been registered with the Chinese Clinical Trial Registry (ChiCTR1900024616, registered on 19 July 2019). The protocol design is based on the guidelines of the Consolidated Standards of Reporting Trials and Standard Protocol Items: Recommendations for Interventional Trials (SPIRIT) (see Additional file 1 SPIRIT 2013 checklist).

### Trial setting

The trial will be conducted in 4 centres, namely, the First Affiliated Hospital of Guangzhou University of Chinese Medicine, the Fifth Affiliated Hospital of Guangzhou University of Chinese Medicine, Guangdong Second Provincial General Hospital, and Pingshan District Peoples’ Hospital of Shenzhen. The clinical investigators from each centre will be responsible for screening eligible participants.

### Participants and recruitment

Participants are being recruited via bulletin board advertisements, acupuncture clinics, and physician referrals from respiratory clinics in 4 centres. The recruitment information mainly includes eligibility criteria and contact details. A well-trained investigator in the acupuncture department will be responsible for the recruitment of participants. Patients meeting the eligibility criteria will be invited to the clinical research centre for further examinations (e.g. pulmonary function test, routine blood test, routine urine test, heart rate, temperature, breath rate and pulse), which will be completed within 1 day.

### Randomization and allocation concealment

Random permuted blocks will be generated by Strategic Applications Software (SAS, version 9.2, SAS Institute, Inc., Cary, USA) and performed by the Key Unit of Methodology in Clinical Research in Guangdong Provincial Hospital of Chinese Medicine. The allocation to the SHP group and to the placebo group will be made in a 1:1 ratio. An independent researcher will prepare a treatment card with a serial number and one of two group numbers on it. Group 1 and group 2 will represent the SHP and placebo groups, respectively. This researcher will attach a label on the tube with ‘group 1’ or ‘group 2’. Participants will be given treatment cards from independent researchers to ensure adequate concealment.

### Blinding

Except for the independent researchers, all participants, acupuncturists, operational assistants, nurses, data managers, and statisticians will be blinded to the treatment allocations until the end of the study. Operational assistants will prepare the patches from tubes labelled with the same name on the treatment card. The placebo patch will be identical in appearance to the SHP. In addition, acupuncturists and operational assistants will be instructed not to communicate with participants about the possibility of their allocation.

To ensure blinding, participants will be required to wait for 120 min in a room and to tear off their treatment patches while facing away from the nurses. In addition, nurses will be instructed not to communicate with participants about the possibility of their allocation.

### Inclusion criteria

The inclusion criteria are as follows:

1. Aged 18–75 years (either sex)
2. Patients with BA of chronic persistence (symptoms appear weekly) or those in clinical remission (maintaining the absence of symptoms for over 1 year)
3. Deficiency of lung and kidney qi syndrome (wheezing, shortness of breath, light and tender tongue with white fur, thin pulse, backache, soft knees, tinnitus, chills and cold limbs, tiredness) [21].
4. Mild to moderate BA (FEV1 ≥ 60%, peak expiratory flow (PEF) variation rate > 20%)
5. Voluntary participation in the study

### Exclusion criteria

The exclusion criteria are as follows:

1. BA with acute exacerbation or in an intermittent state (frequency of symptoms < once a week, FEV1 ≥ 80%, and PEF variation rate < 20%) and/or of severe duration (symptoms appear daily, FEV1 < 60%, and PEF variation rate > 30%)
2. Airflow obstruction disorders, such as chronic obstructive pulmonary disease (COPD), bronchiectasis, and pulmonary tuberculosis
3. Serious health conditions, such as severe cardiovascular and cerebrovascular diseases and liver or kidney diseases (routine blood test, routine urine test, heart rate, temperature, breath rate, and pulse will be examined for safety screening)
4. Mental disorders, such as depression disorder, anxiety disorder, and schizophrenia
5. Allergy to the test ingredients, or blistering, sputum, or damage to the skin at acupoint sites
6. Pregnant women and breastfeeding mothers

### Interventions

#### SHP group

The complete formula for the SHP includes *Angelica dahurica* 1 g (g), *Asarum* 2 g, *Pinellia* 1.5 g, *Corydalis* 0.5 g, *Euphorbia kansui* 1 g, *Brassica alba* 2 g, *Ephedra* 0.6 g, *Arisaema consanguineum* 0.8 g, *Atractylodes macrocephala* 1 g, and musk 0.1 g. The herbs are processed into powder and mixed with fresh ginger juice to create the SHP ointment. The SHP and placebo are manufactured by the Pharmaceutical Preparation Department in the First Affiliated Hospital of Guangzhou University of Chinese Medicine to meet the requirements of the regulatory guidance issued by the China Food and Drug Administration.

**Fig. 3:**
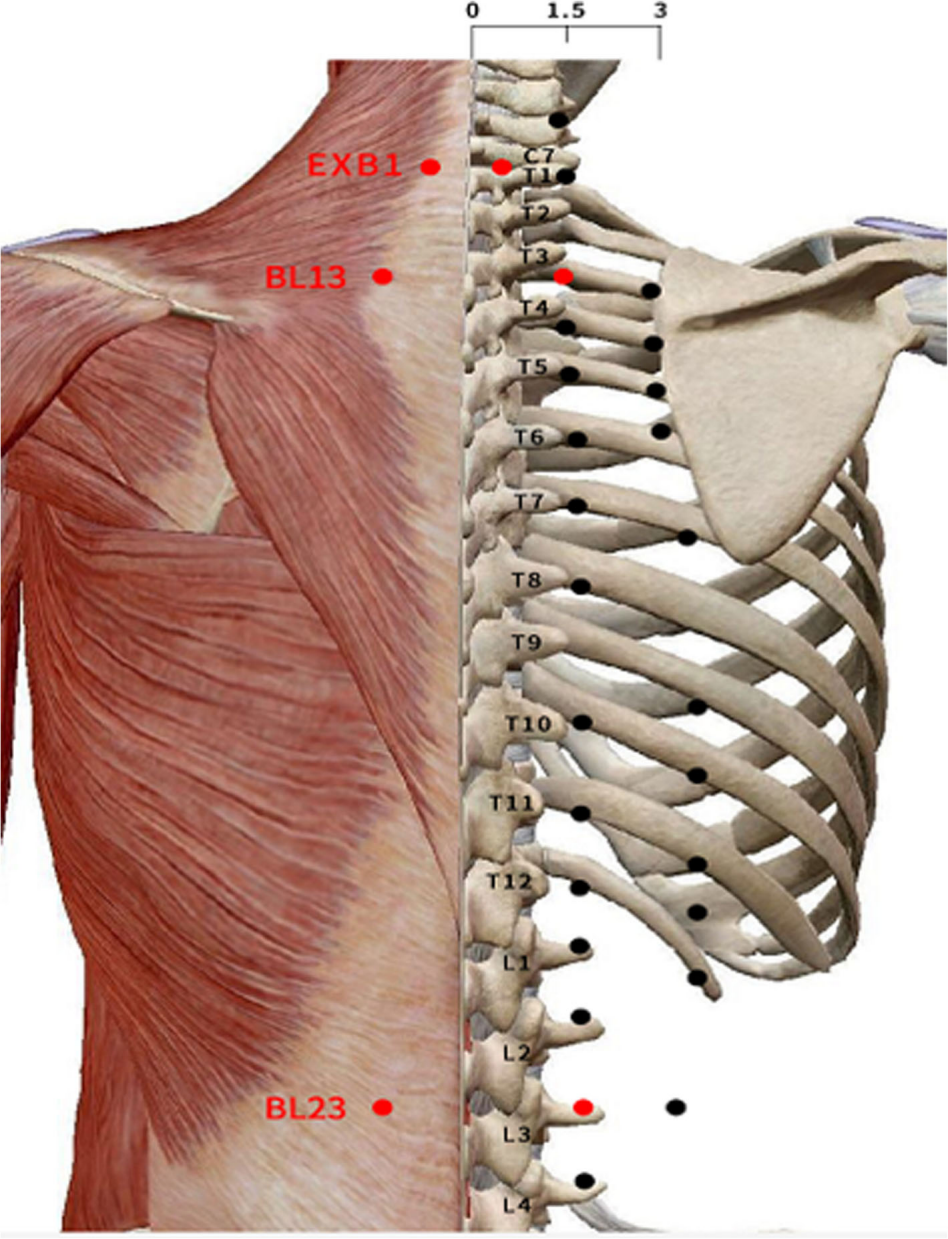
Acupuncture points for both groups

The selected acupoints are Feishu (BL13), Dingchuan (EX B1), and Shenshu (BL23) on both sides, for a total of 6 acupoints. The locations of the acupoints are shown in Fig. 3 according to the World Health Organization Standard Acupuncture Locations.

Participants receive an SHP through the following procedure: (1) each participant is asked to expose their back. (2) Two grams of ointment are squeezed by operational assistants onto a circular medical-proof fabric, with each piece of SHP being 1 cm in diameter. (3) One piece of the SHP is attached to each acupoint by an acupuncturist in each centre. (4) Participants are then asked to wait in a room for 120 min, and nurses are required to carefully observe them. If a participant feels any unbearable pain or a burning sensation, the SHP is to be removed immediately by the nurses. The exact treatment duration for each participant is recorded on the case report form (CRF).

#### Placebo group

The placebo ointment is similar to the SHP in physical properties such as appearance, colour, dosage form, weight, and gas. It is composed of buckwheat flour, caramel, and water. The acupoints, dosage of ointment, medical proof fabric, and procedures are the same as those in the SHP group.

There are 3 TSs in 2020, and the exact dates for each session are calculated according to the Chinese Lunar Calendar (Table 1).

**Table 1:** Date and acupoints for each treatment session in 2020 in the study

Year		1st TS	2nd TS	3rd TS
2020	dates	16 July (Initial Sanfu)	26 July (Middle Sanfu)	15 August (Final Sanfu)
	acupoints	BL13 (bilateral)	BL13 (bilateral)	BL13 (bilateral)
		BL23 (bilateral)	BL23 (bilateral)	BL23 (bilateral)
		EX B1 (bilateral)	EX B1 (bilateral)	EX B1 (bilateral)

#### Concomitant medications

Participants will be allowed to take budesonide and formoterol fumarate powder for inhalation (AstraZeneca AB, Sweden), 4.5 μg per dose, twice a day. For participants undergoing an acute attack, salbutamol sulphate inhalation aerosol (GlaxoSmithKline Australia Pty, Ltd.) will be given at 100 to 200 μg per dose, repeating inhalation every 4 to 8 h if necessary.

Participants will be requested not to receive any other treatments, such as complementary treatments (e.g. moxibustion, herbal medicine, and massage) or other medications, throughout this study. The use of all drugs, if any, will be documented in detail in the CRF.

### Trial outcomes

#### Primary outcome

The primary outcome is the change in the FEV1 from baseline to the end of the 3 TSs. FEV1 will be performed by the Pulmonary Function Test System (MasterScreen, CareFusion Germany 234 GmbH) in the Pulmonary Function Examination Room of the First Affiliated Hospital of Guangzhou University of Chinese Medicine. Clinically, an FEV1 value less than 80% of the expected value indicates pulmonary dysfunction [22].

The efficacy evaluation is as follows:

1. Clinically completely controlled: asthma attacks are completely relieved, with FEV1 improved by more than 35%;
2. Significantly controlled: asthma attacks are significantly relieved, with FEV1 improved by 25–35%;
3. Partly controlled: FEV1 improved by 15–24%;
4. Uncontrolled: no improvement in asthma attacks or FEV1 measurement.

Total treatment efficiency = (clinically complete controlled number + significant controlled number + partly controlled number)/total number ×  100%.

### Secondary outcome

#### Questionnaire test

As secondary outcomes, we will use the Asthma Quality of Life Questionnaire (AQLQ) [23] and the Asthma Control Test (ACT) [24] at the 2nd and 3rd TSs and at the 12th and 24th weeks compared with baseline.

The AQLQ contains 35 questions with four domains (symptoms, activity limitation, emotional function, and environmental stimuli) as a measure of quality of life for adult patients with asthma. The items are self-assessed by participants using a seven-point scale (1 = completely restricted; 2 = extremely restricted; 3 = severely restricted; 4 = moderately restricted; 5 = mildly restricted; 6 = rarely restricted; 7 = unrestricted). The scores for each question are averaged to produce an overall score. The study will compare the mean change scores from baseline at each time point, using the mean scores from each group.

The ACT is a widely used, validated questionnaire for the assessment of asthma control [25]. The ACT contains 5 questions, and each allows a response on a 1 to 5 scale, with 5 representing no problems at all. Five points are added to the total ACT score (< 20: poorly controlled; 20–24: partly controlled; 25: well controlled asthma).

In addition, participants will be asked to complete an Asthma Long-term Follow-up Scale (ALFS) [26] at the 12th and 24th weeks of follow-up. The frequency and severity of symptoms, occurrence time, FEV1, and use of medications (name, dose and date) will be recorded in detail on the ALFS to assess the safety in each group.

#### Laboratory indicators

##### Airway inflammatory factors

The levels of airway inflammatory factors (IL-5, IL-13, IL-23, IL-25, TSLP) will be assessed at baseline and at the end of the 2nd and 3rd TSs. Five millilitres of antecubital vein blood taken from participants on an empty stomach will be collected by trained clinical staff in each centre. Sera will be obtained by coagulation and centrifugation of blood samples for 20 min at 1000*g* at room temperature and then aliquoted and frozen at − 80 °C before analysis. To determine the antibody response, the samples will be tested by a double-antigen sandwich enzyme-linked immunosorbent assay (ELISA) [27, 28]. All samples will be measured in duplicate and averaged.

##### Tracheal smooth muscle cell regulatory proteins

Five millilitres of serum and urine of participants will be obtained from participants at baseline and at the end of the 2nd and 3rd TSs. ELISA will be used to measure the levels of tracheal smooth muscle cell regulatory proteins (Metallothionein-2, Transgelin-2) in serum and urine. Urine samples will be obtained by centrifugation of urine at 1000*g* for 20 min; then, particulates will be removed immediately, and the sample will be stored at − 80 °C.

ELISA will be performed according to the kit instructions (Wuhan Youersheng Trading Co., Ltd., China) at Lingnan Medical Research Centre in the First Affiliated Hospital of Guangzhou University of Chinese Medicine.

### Safety aspects

#### Adverse events

Any adverse events (AEs) occurring to participants during the treatment period and follow-up, regardless of whether they are associated with the intervention, will be assessed and recorded at any time. Some AEs, such as local itching, redness, and blisters, may be related to the SHP [29]. After every treatment, participants will be required to wait for 120 min in a room for medical observation. To ensure blinding, their patches will be torn off by the nurses. During this period, if participants feel slight burning, tingling, or redness in the local skin, nurses will remove the treatment patches within 120 min. If small blisters occur, the nurses will carefully prick the blisters with a disinfection needle and then cover them with sterile gauze to prevent infection. All details of AEs will be recorded on CRFs.

Severe adverse events (SAEs) or adverse drug reactions (ADRs) include repeated episodes of BA, admissions for treatment, prolonged hospital stays, life-threatening conditions, and death. SAEs must be reported to the Ethics Committee of the First Affiliated Hospital of Guangzhou University of Chinese Medicine within 24 h. In the event of an emergency medical situation, the individual’s randomization code and group allocation can be identified via a standard operational procedure.

### Statistical methods

#### Sample size

The sample size was calculated based on the primary outcome measure by PASS11.0 (Power Analysis and Sample Size, NCSS, LLC., Kaysville, UT, USA). The power was set at 90% and calculated based on two-sided 95% confidence intervals. Based on a mean score of 79.41 with a standard deviation (SD) of 13.87 in the SHP group and a mean score of 59.61 (SD 11.52) in the placebo group after treatments [30], the sample size was determined to be 30 for each group. Considering a 15% drop-out rate, 36 patients will be included in each group. Finally, we will require a sample size of 72 patients with BA in this study.

#### Data analysis

The final data will be double-entered by two statisticians and analysed by PASW Statistic 24.0 (IBM SPSS, Inc., Armonk, NY, USA). (1) All analyses will be based on the intention-to-treat principle. Thus, multiple imputations will be used to address the missing data. Two-tailed *p* values < 0.05 will be considered statistically significant. (2) For continuous variables, all data will first be tested for normality. The *t* test will be used for the fitted data, while the rank sum test will be used for the non-fitted data. Continuous variables will be described as the mean ± standard deviation (¯x ± SD), maximum, and minimum. (3) Repeated measures will be used to compare the value differences of several continuous observations. (4) For categorical variables, the chi-square test or Fisher’s exact test will be used to examine the between-group differences, and percentages and frequencies will be presented to describe the effect size.

### Quality supervision and management

Before recruitment, the whole research team at the 4 centres, including acupuncturists, operational assistants, and data statisticians, will be required to attend a training workshop to ensure strict adherence to the study protocol. They will be provided with a written protocol and standard operating procedure documents. During the study period, clinical trial collaboration meetings will be held regularly to check the progress of the research.

All data collected from participants and modifications will be clearly documented on CRFs. The last-visit-carried-forward method will be used to handle the missing data. Data will be entered using the double-entry method and reported to the clinical trial centre. Data managers will recheck the data before logging them and will promptly notify the research team if any discrepancy is found.

The clinical trial will be suspended or discontinued if the safety of any participant is compromised or if any SAEs are not reported within the prescribed time. If the trial is suspended or discontinued, the participants, the project funder, and the ethics committee will be informed of the reason.

### Ethics and dissemination

#### Patient consent and dissemination policy

This protocol has been registered with the Chinese Clinical Trial Registry. In the first visit, each participant will be informed in detail about the study procedures and provided with enough time to decide whether to sign the written consent before randomization. All blood and urine samples obtained from the participants will be destroyed after the trial. Participants can withdraw their informed consent at any time during the study. Participants who experience severe adverse reactions or irreversible injuries as a result of the trial will be reasonably compensated according to the circumstances.

#### Access to data and confidentiality

Every precaution will be taken to respect the privacy of participants in the conduct of the study. Only research team members will have access to the research data. All data will be kept strictly confidential during the study. Information will be stored in password-protected files and held independently by the staff of the Key Unit of Methodology in Clinical Research in Guangdong Provincial Hospital of Chinese Medicine. In the course of monitoring data quality and adherence to the study protocol, the monitors will refer to medical records at 4 centres. On completion of the study, participants will receive a personal summary of the data collected in the study.

## Discussion

To the best of our knowledge, this will be the first clinical study to investigate the anti-inflammatory mechanism and long-term efficacy of SHP for BA at 4 centres. Compared with previous studies [17, 18, 31], this trial has a larger participant pool, clearer diagnostic criteria for the classification of BA, a more rigorous methodology, and objective indicator detection. We are focusing on lung function, quality of life, and airway inflammation during the treatments and long-term follow-up period.

BA can be divided into acute attack, chronic duration, and clinical remission based on the duration and severity of symptoms [21]. However, previous trials evaluating the effect of SHP have examined BA without clarifying the differences between 3 stages.

Taking real clinical practice into account, we are focusing on patients with BA of chronic duration and clinical remission, with a severity ranging from mildly to moderately persistent. Many previous studies of SHP results were mostly limited to the evaluation of clinical efficacy and the summary of their experience [32]. Thus, further research is needed to explore the possible mechanisms.

The pathogenesis of BA is very complicated. Current studies mainly focus on three targets: airway hyperresponsiveness, airway inflammation, and tracheal smooth muscle spasm [33]. However, airway inflammation is considered to be the primary component contributing to the heterogeneity and severity of airway disorders [34]. It is characterized by increased infiltration of various inflammatory cells, including airway inflammatory cells and structural cells (e.g. eosinophils, neutrophils, mast cells, smooth muscle cells, T lymphocytes, and airway epithelial cells) and cell components [21]. For example, interleukin 5 (IL-5), interleukin 13 (IL-13), and interleukin 25 (IL-25) are thought to be essential to the allergic asthmatic response [35]. Constitutive overexpression of IL-13 shows it to be essential for airway hyperreactivity, mucus production, and inflammation [36]. In addition, thymic stromal lymphopoietin (TSLP) can induce airway inflammation in asthma [37]. The selected acupoints in this trial are Feishu (BL13), Dingchuan (EX B1), and Shenshu (BL23). Previous studies showed that EX B1 in combination with BL13 had a significant effect on the treatment of asthma-related cough [38], and BL23 could enhance lung ventilation [39]. Therefore, we suspect that SHP at acupoints on BL13, EX B1, and BL23 may be related to changes in inflammatory factors, including IL-5, IL-13, IL-23, IL-25, and TSLP.

However, bronchoconstriction causes airway narrowing and generates significant morbidity. Transgelin-2, a smooth muscle marker, is a unique actin-binding protein involved in asthma and cancer [40, 41]. Metallothionein-2 (MT-2) is an isoform of the metallothionein family that has been reported to regulate calcium ion concentrations in smooth muscle cells. A previous study showed that MT-2 protein levels were more than 50% lower in asthmatic lung tissue than in control samples [42]. However, the relationship between SHP and MT-2 expression during asthma is still unknown. In this study, changes in MT-2 and Transgelin-2 in serum and urine will be detected. If their levels rise and BA symptoms decrease, we may speculate that the SHP possibly works by reducing bronchospasm.

This study also has limitations. AEs of the SHP may be allergic skin reactions, such as local skin itching, redness, and blisters. In some cases, AEs may increase the drop-out rate. However, several measures will be taken throughout the study. First, a previous allergic history would be required before recruitment. AEs will be described in detail on the consent forms provided at the first visit. Second, the degree of skin allergic reactions will be evaluated according to the standards of the *Guidelines for the Research of New Drugs in Traditional Chinese Medicine* (*Pharmaceutics*, *Pharmacology*, *Toxicology*) by experienced acupuncturists. Third, nurses will carefully observe the participants after SHP treatment in the room for 120 min and record findings.

In summary, this protocol describes a randomized controlled trial conducted in 4 centres to assess the efficacy and safety of an SHP applied at acupoints in the treatment of BA. The results of this trial will provide high-quality evidence of the clinical effects of the SHP in alleviating asthma symptoms and improving quality of life among BA patients. Moreover, tracheal smooth muscle cell regulatory proteins (MT-2 and Transgelin-2) and airway inflammatory factors (IL-5, IL-13, IL-23, IL-25, and TSLP) will be tested. If the efficacy and safety are recognized, the mechanism of SHP in reducing airway inflammation and relaxing bronchospasm will be further explored. Thus, doctors will have more treatment options for BA.

### Trial status

The participants are beginning to be recruited for this study (protocol version 2.0, 19 July 2019). The study will run from May 2020 to December 2021.

## Supplementary information

**Additional file 1.** SPIRIT 2013 Checklist: Recommended items to address in a clinical trial protocol and related documents.

**Additional file 2.**

## Data Availability

Data from this randomized controlled study are unavailable at the time of publication. Individual participant data are available upon request.

## Abbreviations

BA: Bronchial asthma
SHP: Sanfu herbal patch
TS: Treatment session
ICS: Inhaled corticosteroids
LTRA: Leukotriene receptor antagonists
RCT: Randomized controlled trial
SPIRIT: Standard Protocol Items: Recommendations for Interventional Trials
SAS: Strategic Applications Software
PEF: Peak expiratory flow
COPD: Chronic obstructive pulmonary disease
AQLQ: Asthma Quality of Life Questionnaire
ACT: Asthma Control Test
ALFS: Asthma Long-term Follow-up Scale
ELISA: Enzyme-Linked Immunosorbent Assay
AEs: Adverse events
SAEs: Severe adverse events
ADRs: Adverse drug reactions
SD: Standard deviation
MT-2: Metallothionein-2
TSLP: Thymic stromal lymphopoietin

## References

[1] Papadopoulos NG, Arakawa H, Carlsen KH, Custovic A, Gern J, Lemanske R, Le Souef P, Makela M, Roberts G, Wong G (2012). International consensus on (ICON) pediatric asthma. Allergy.

[2] Genuneit J, Seibold AM, Apfelbacher CJ, Konstantinou GN, Koplin JJ, La Grutta S, Logan K, Flohr C, Perkin MR (2017). The state of asthma epidemiology: an overview of systematic reviews and their quality. Clin Transl Allergy.

[3] Anandan C, Nurmatov U, van Schayck OC, Sheikh A (2010). Is the prevalence of asthma declining? Systematic review of epidemiological studies. Allergy.

[4] Wang CZ. Improve the state of bronchial asthma control, need to value the long-term management of patients. Chin J Lung Dis. 2013. 10.3877/cma.j.issn.1674-6902.2013.04.001.

[5] Kim RY, Pinkerton JW, Gibson PG, Cooper MA, Horvat JC, Hansbro PM (2015). Inflammasomes in COPD and neutrophilic asthma. Thorax.

[6] Chipps BE, Zeiger RS, Dorenbaum A, Borish L, Wenzel SE, Miller DP, Hayden ML, Bleecker ER, Simons FE, Szefler SJ (2012). Assessment of asthma control and asthma exacerbations in the epidemiology and natural history of asthma: outcomes and treatment regimens (TENOR) observational cohort. Curr Respir Care Rep.

[7] Graff S, Demarche S, Henket M, Paulus V, Louis R, Schleich F (2019). Increase in blood eosinophils during follow-up is associated with lung function decline in adult asthma. Respir Med.

[8] Matsushita S, Yamashiro T, Matsuoka S, Yagihashi K, Nakajima Y (2019). The association between bronchial wall CT attenuation and spirometry in patients with bronchial asthma. Acad Radiol.

[9] Grosso A, Pesce G, Marcon A, Piloni D, Albicini F, Gini E, Marchetti P, Battaglia S, Ferrari M, Fois A (2019). Depression is associated with poor control of symptoms in asthma and rhinitis: a population-based study. Respir Med.

[10] Alvarez GG, Schulzer M, Jung D, Fitzgerald JM (2005). A systematic review of risk factors associated with near-fatal and fatal asthma. Can Respir J.

[11] Bateman ED, Hurd SS, Barnes PJ, Bousquet J, Drazen JM, FitzGerald. et al, Global strategy for asthma management and prevention: GINA executive summary. Eur Respir J. 2008. 10.1183/09031936.00138707. 10.1183/09031936.00138707 18166595

[12] Gong SK, Gao D, Fang H. A review on treating bronchial asthma in the integrative medicine. Clin J Chin Med. 2018. 10.3969/j.issn.1674-7860.2018.05.061.

[13] Yin KS. Problems and countermeasures in asthma medication. J Clin Med Pract. 2008. 10.3969/j.issn.1672-2353.2008.05.004.

[14] Zhou F, Yang D, Lu JY, Li YF, Gao KY, Zhou YJ, Yang RX, Cheng J, Qi XX, Lai L (2015). Characteristics of clinical studies of summer acupoint herbal patching: a bibliometric analysis. BMC Complement Altern Med.

[15] Yang XC, Yin T, Gao Q, Kong LJ. The immunomodulatory effect of acupoint application for childhood asthma: a systematic review and meta-analysis. Evid Based Complement Alternat Med. 2015. 10.1155/2015/896247. 10.1155/2015/896247 PMC4426892 26000027

[16] Zhang L (1990). Zhang shi yi tong.

[17] Xiaqiu W, Jin P, Guoqin L, Wei Z, Guangxia L, Baoyan L (2015). Efficacy evaluation of summer acupoint application treatment on asthma patients: a two-year follow-up clinical study. J Tradit Chin Med.

[18] RanBang M, HyunKim J, YeonMin S (2017). Clinical effectiveness of the Sanfu herbal patch at acupoints for respiratory diseases including otitis media in children: a pilot before-and-after study. Eur J Integr Med.

[19] Cai JX, Ye DL, Liang M. Effect of Ke Chuan San Fu Tie on IL-4, IFN-γ in children patients in the asthma remission. Liaoning J Tradit Chin Med. 2008. 10.3969/j.issn.1000-1719.2008.06.006.

[20] Zhou F, Liang N, Maier M, Liu JP (2017). Sanfu acupoint herbal patching for stable asthma: a systematic review and meta-analysis of randomised controlled trials. Complement Ther Med.

[21] Asthma Study Group of Chinese Thoracic Society 2016 asthma management and prevention guidelines (In Chinese). Chin J Tuberc Respir Dis. 2016; doi:10.3760/cma.j.issn.1001-0939.2016.09.007.

[22] Zheng JP, Gao Y (2010). Practical guide to pulmonary function testing.

[23] Everhart RS, Smyth JM, Santuzzi AM, Fiese BH (2010). Validation of the Asthma Quality of Life Questionnaire with momentary assessments of symptoms and functional limitations in patient daily life. Respir Care.

[24] Hasegawa T, Koya T, Sakagami T, Kagamu H, Arakawa M, Gejyo F, Narita I, Suzuki E, Group tNATS (2013). The Asthma Control Test, Japanese Version (ACT-J) as a predictor of Global Initiative for Asthma (GINA) guideline-defined asthma control: analysis of a questionnaire-based survey. Allergol Int.

[25] Ramakrishnan K, Lee LK, Safioti G (2019). Asthma Control Test (ACT) scores correlate with health-related quality of life (HRQoL) in patients with asthma. J Allergy Clin Immunol.

[26] Zarqa A, Glattre DC, Suppli UC (2013). Long-term mortality among adults with asthma. Chest.

[27] Jia LQ, Ge ZL, Jin TY, Bao YF, Ou GW. Improvement and application of a competitive ELISA for detection of metallothionein in human urine. J Environ Occup Med. 2010. 10.13213/j.cnki.jeom.2010.03.020.

[28] Watcharatanyatipa K, Boonmoha S, Chaichounb K, Songsermc T, Woratantia M, Dharakula T (2010). Multispecies detection of antibodies to influenza A viruses by a double-antigen sandwich ELISA. J Virol Methods.

[29] Zhang XY, Wu L, Liu ZY, Guo H, Ou HY, Li YK, et al. Clinical observation of vesiculation moxibustion for allergic rhinitis due to deficient cold of lung-qi. Shanghai J Acupunct Moxibustion. 2014. 10.13460/j.issn.1005-0957.2014.10.0906.

[30] Lu YM. Summary of 35 cases of relief period of bronchial asthma treated with Sanfu acupoint patch. Hunan J Tradit Chin Med. 2011. 10.16808/j.cnki.issn1003-7705.2011.03.009 (In Chinese).

[31] Zhu L, Zhang W, Wong V, Eric Z, Lao L, Lo K, et al. Randomized trial of acupoints herbal patching in Sanfu days for asthma in clinical remission stage. Clin Transl Med. 2016. 10.1186/s40169-016-0084-7. 10.1186/s40169-016-0084-7 PMC4742458 26846122

[32] Zhao JP, Zhang QY, Qi X, Zhao X, Guan F, Li YJ, et al. Review on treatment of traditional Chinese medicine acupoint sticking on respiratory diseases. Chin Arch Tradit Chin Med. 2017. 10.13193/j.issn.1673-7717.2017.07.039.

[33] Fahy JV (2015). Type 2 inflammation in asthma--present in most, absent in many. Nat Rev Immunol.

[34] Mukherjee M, Sehmi R, Nair P (2014). Anti-IL5 therapy for asthma and beyond. World Allergy Organ J.

[35] Barlow JL, Bellosi A, Hardman CS, Drynan LF, Wong SH, Cruickshank JP, et al. Innate IL-13–producing nuocytes arise during allergic lung inflammation and contribute to airways hyperreactivity. J Allergy Clin Immunol. 2012. 10.1016/j.jaci.2011.09.041. 10.1016/j.jaci.2011.09.041 22079492

[36] Halim TY, Steer CA, Matha L, Gold MJ, Martinez-Gonzalez I, McNagny KM, McKenzie AN, Takei F (2014). Group 2 innate lymphoid cells are critical for the initiation of adaptive T helper 2 cell-mediated allergic lung inflammation. Immunity.

[37] Yu G, Zhang Y, Wang X, Sai L, Bo C, Yeo AJ, Lavin MF, Peng C, Jia Q, Shao H (2019). Thymic stromal lymphopoietin (TSLP) and toluene-diisocyanate-induced airway inflammation: alleviation by TSLP neutralizing antibody. Toxicol Lett.

[38] Fan L, Wang Y, Yin LM, Xu YD, Ran J, Wang WQ, et al. Verification of the origin and development of the nomenclature and location of point Dingchuan (Ex-B1). Shanghai J Acupunct Moxibustion. 2015. 10.13460/j.issn.1005-0957.2015.02.0167.

[39] Deng RS, Huang H, Wu XW, Wen ZJ. Effects of moxibustion of Zusanli and Shenshu on function of pulmonary ventilation of tennis athletes. J ZhanJiang Norm Coll. 2010. 10.3969/j.issn.1006-4702.2010.03.021.

[40] Yin LM, Ulloa L, Yang YQ (2019). Transgelin-2: biochemical and clinical implications in cancer and asthma. Trends Biochem Sci.

[41] Matsui TS, Ishikawa A, Deguchi S (2018). Transgelin-1 (SM22alpha) interacts with actin stress fibers and podosomes in smooth muscle cells without using its actin binding site. Biochem Biophys Res Commun.

[42] Zhou D-D, Ran J, Li C-C, Lu J, Zhao Q-Y, Liu X-Y, et al. Metallothionein-2 is associated with the amelioration of asthmatic pulmonary function by acupuncture through protein phosphorylation. Biomed Pharmacother. 2019. 10.1016/j.biopha.2019.109785. 10.1016/j.biopha.2019.109785 31874444

