# Supplementary material for: Sanfu herbal patch applied at acupoints in patients with bronchial asthma: study protocol for a randomized controlled trial
Source: Trials. 2020 Jul 29;21:684. doi: 10.1186/s13063-020-04604-8 (PMC7389380; doi:10.1186/s13063-020-04604-8)
Supplement: Supplementary file 2 — Additional file 2. [file 13063_2020_4604_MOESM2_ESM.pdf]

关于认可伦理审批件的声明

本单位参与由广州中医药大学第一附属医院资助的课题：基于冬病夏治理论进行天灸药物贴敷防治支气管哮喘的临床规范化研究及机制探讨（课题编号：2019IIT04）。该课题已通过组长单位伦理委员会审批（编号：ZYYECK[2019]049）。本单位承诺该伦理审批件适用本单位开展此研究，并将获得所有研究参与者的知情同意。

负责人：

参与单位：广东省第二人民医院

Statement on recognition of ethical approval letter

This unit participated in a project funded by the First Affiliated Hospital of Guangzhou University of Chinese Medicine: Clinical standardized research and mechanism research on the prevention and treatment of bronchial asthma based on the theory of treating winter disease in summer (number: 2019IIT04). This subject has been approved by the ethics committee of the leader unit (number: ZYYECK [2019] 049). We promises that the ethical approval is applicable to the unit to carry out this research and will obtain the informed consent from all study participants.

Principal signature

Participating centre: Guangdong Second Provincial General Hospital

关于认可伦理审批件的声明

本单位参与由广州中医药大学第一附属医院资助的课题：基于冬病夏治理论进行天灸药物贴敷防治支气管哮喘的临床规范化研究及机制探讨（课题编号：2019IIT04）。该课题已通过组长单位伦理委员会审批（编号：ZYYECK[2019]049）。本单位承诺该伦理审批件适用本单位开展此研究，并将获得所有研究参与者的知情同意。

参与单位：广东省第二中医院

Statement on recognition of ethical approval letter

This unit participated in a project funded by the First Affiliated Hospital of Guangzhou University of Chinese Medicine: Clinical standardized research and mechanism research on the prevention and treatment of bronchial asthma based on the theory of treating winter disease in summer (number: 2019IIT04). This subject has been approved by the ethics committee of the leader unit (number: ZYYECK [2019] 049). We promises that the ethical approval is applicable to the unit to carry out this research and will obtain the informed consent from all study participants.

Participating centre:

The Fifth Affiliated Hospital of Guangzhou University of Chinese Medicine

广东省第二中医院 针灸康复科

关于认可伦理审批件的声明

本单位参与由广州中医药大学第一附属医院资助的课题：基于冬病夏治理论进行天灸药物贴敷防治支气管哮喘的临床规范化研究及机制探讨（课题编号：2019IIT04）。该课题已通过组长单位伦理委员会审批（编号：ZYYECK[2019]049）。本单位承诺该伦理审批件适用本单位开展此研究，并将获得所有研究参与者的知情同意。

参与单位：深圳市坪山区人民医院

Statement on recognition of ethical approval letter

This unit participated in a project funded by the First Affiliated Hospital of Guangzhou University of Chinese Medicine: Clinical standardized research and mechanism research on the prevention and treatment of bronchial asthma based on the theory of treating winter disease in summer (number: 2019IIT04). This subject has been approved by the ethics committee of the leader unit (number: ZYYECK [2019] 049). We promises that the ethical approval is applicable to the unit to carry out this research and will obtain the informed consent from all study participants.

Participating centre:

Pingshan District Peoples' Hospital of Shenzhen (Official seal)
